# How does users' interest influence their click behavior?: evidence from Chinese online video media

**DOI:** 10.3389/fpsyg.2023.1101396

**Published:** 2023-07-06

**Authors:** Dongqi Li, Zihuang Tang, Nan Zhao

**Affiliations:** ^1^CAS Key Laboratory of Behavioral Science, Institute of Psychology, Chinese Academy of Sciences, Beijing, China; ^2^Department of Psychology, University of Chinese Academy of Sciences, Beijing, China

**Keywords:** interest, click behavior, online video media, linear regression, mediation analysis

## Abstract

Interest is one of the main factors motivating an individual's behavior, and its effect in the learning process has been widely confirmed in educational psychology. The purpose of this study was to explore the influence of individual interest, topic interest and situational interest on the user's video click behavior in the online video browsing situation. We constructed an online experiment in which each participant was asked to use questionnaires to assess their responses to video categories, titles, and covers from the video-sharing website, Bilibili. Based on these responses, we obtained individual interests, topic interests, situational interests, and click behavior of the participants toward the videos. Correlation, regression and mediation analyses were conducted to explore the effects and mechanisms of the three interests on click behavior. The results found: (1) individual interest may have a positive but relatively weaker effect on click behavior, and (2) topic interest and situational interest positively predicted click behavior in all categories. The mediation analysis found: (1) in the otomads and fashion categories, the effect of individual interest on click behavior was partially mediated by topic and situational interest, and (2) in the anime, digits, life, dance, music, game, entertainment, and knowledge categories, the effect of individual interest on click behavior was fully mediated by topic interest and situational interest. These results revealed the facilitating effects and different effect modes of individual, topic, and situational interest on click behavior. These findings shed light on the influence mechanism of interests on video click behavior in different video categories and provide new insights into related applications such as recommender.

## 1. Introduction

When a user is browsing an online video-sharing platform, how does the information they receive prompt them to click and watch a certain video? This is a valuable question for both researchers and practitioners. In the field of information technology, recommendation algorithms can use the user's past click records as a label to build models to select new videos that are more likely to be clicked by the user (Lv, 2017; Inaba and Takahashi, 2018; Kushima et al., 2019). Although effective in triggering users' click behavior, recommendation models are usually incomprehensible, as they are not based on the psychological process from the online stimuli to the click behavior. From the perspective of psychology, a user clicks on a certain video because they have the intention of paying more attention to it and exploring it more, and this intention is interest.

As a psychological term, interest is defined as a motivational variable (Inaba and Takahashi, 2018) that draws attention to specific objects or stimuli and directs individuals' involvement in specific activities (Renninger and Hidi, 2016). Interest has particularly been discussed in previous studies of educational psychology, in which it can be classified into three categories: individual interest, situational interest, and topic interest. Different categories of interest were often triggered by different stimuli or information.

Individual interests are relatively enduring preferences of individuals toward certain domains or activities over time (Hidi and Baird, 1988; Hidi, 1990; Hidi and Renninger, 2006). It is related to the individual's characteristics and is based on their existing knowledge structure and experiences. Thus, individual interests are often tied to specific categories of content (Trautwein et al., 2015). Different from individual interest, situational interest is a psychological state that is induced by a specific situational stimulus (Schank et al., 1979; Kintsch, 1986; Hidi and Baird, 1988). In contrast to individual interest, it is immediate and spontaneous and is influenced by external conditions. Situational interest mainly depends on the characteristics of the thing itself and the subject's feelings about it (Hidi and Anderson, 1992). Topic interest is a third form of interest that can be distinguished from the two aforementioned interests (Wijnia et al., 2014) and refers to the interest in a topic elicited by a word or a phrase (Hidi and Berndorff, 1998). It can, therefore, be considered an anticipatory response to a topic that is influenced by both individual and situational interests (Renninger, 2000; Hidi, 2006; Mason et al., 2008). Some researchers have argued that topic interest is a specific expression of individual interest (Schiefele, 1996; Schiefele and Krapp, 1996), but more scholars believe that topical interest is shaped by a combination of both personal and situational factors (Wade et al., 1999; Renninger, 2000).

All these three categories of interest could promote explorative behaviors to some extent, while the processes and mechanisms of this promotion do not seem to be the same. A lot of research evidence regarding interest comes from studies of learning, and improvements in learning behavior have been found to be facilitated by all three categories of interest. Some studies have shown that individual interest can enhance the learning process by improving the reader's ability to reason about the text and by facilitating deep cognitive processing of the text (Fransson, 1977; Zhang and Zhang, 1996). Individual interest is based on one's knowledge and experience, and its important characteristic is that it comes from individuals' internal needs rather than external factors (Schiefele, 1991). Individual interest means that individuals have a higher accumulation of knowledge and experience in this field, which can play an important role in text reasoning and deep cognitive processing. The facilitation effect of topic interest on learning is reflected in the individual memory process and cognitive processing ability (Soemer and Schiefele, 2019; McIntyre et al., 2021). The motivation of maintaining and exploring a specific subject facilitated individuals' memory and cognition of it. Situational interest has been demonstrated to have a role in promoting individual achievement behaviors (Chen et al., 2001; Huang and Gao, 2013; Zhu and Chen, 2017; Thomas and Kirby, 2020). Unlike individual interests, situational interests depend on the characteristics of external things, such as the learning environment, and how the individual feels about them (Zhu and Chen, 2017; Dorfner et al., 2018; Ribeiro et al., 2018; Grund et al., 2019; Yu et al., 2019; Roure et al., 2020). This means that some characteristics of the perceived object draw the individual's attention to it, which eventually leads to the generation of situational interest and even subsequent exploration behavior. In general, interest has also been proven to significantly determine the act of choices in real life, from simple reading material to an academic task or even a career (Morgan et al., 2001; Yeasin et al., 2006; Shigemitsu and Nittono, 2008; Päßler and Hell, 2012).

Online video browsing may fulfill multiple purposes, while the basic behavior process was highly similar: receiving partial information, triggering motivation for further exploration, and choosing to watch a certain video or not. This production of motivation for further exploration based on the received information also commonly exists in the learning process and could be reasonably interpreted by the framework of interest. When users browse videos on a video-sharing platform such as YouTube and Bilibili, three attributes of the video are first shown to the user: the category, the title, and the cover. The user selects which video to watch just depending on these three attributes and then click on a certain video to watch. The category, or the channel, of a video, implies the type to which it belongs. These types are usually classified according to the video content. A video category represents a certain domain or activity, and the user's interest triggered by the category meets the criterion of individual interest as defined earlier in this article. The title of a video presents the specific theme and topic of the video in the text. As interest in the topic is an immediate anticipatory reaction elicited by reading a title or a short textual description (Schiefele, 1996; Ainley et al., 2002), the user's interest triggered by the video title is the topic interest. Video covers are usually attractive images relative to the video, which provide a decorative visual situation beyond the messages obtained from the video category and the video title. Previous studies have shown that decorative pictures in learning materials elicit learners' situational interest, and the relevance, presentation location (Harp and Mayer, 1998; Rowland et al., 2008), and other features of the picture affect this process. It is not surprising that the video cover image triggers the situational interest of the user.

In summary, before the click behavior, three categories of interest can possibly to be induced while browsing the web page of the video site. Although the motivational role of these interests has been widely demonstrated in many real-life tasks, there is still a lack of relevant research revealing the effects of the three categories of interest on video clicks. In previous studies on the influence of interest on video watching, researchers mainly focused on the information processing during the viewing of a specific video (Li et al., 2017). However, there is still little knowledge on how interest drives users to choose a video before watching it. Do the different interests all promote click behavior effectively? Do they play equally important roles in the user's choice of video? The answers to these questions are crucial for understanding the psychological processes and contributing factors of video selection during online browsing. Furthermore, the user's interest in watching a certain video is ultimately triggered by the video elements they received during browsing. Exploring the impact of different interests on users' video selection can provide new insights into the roles of different video elements. In turn, it can provide some guidance on the psychological view of the involvement of recommendation systems.

To reveal the influence of different interests on video selection during online browsing, the present study conducts experiments in an online video browsing context. Our preliminary hypotheses are as follows: (1) all three interests can facilitate the occurrence of click behavior, and (2) the three interests are weighted differently and act in different ways in the click decision. The empirical evidence from these experiments may help us to better understand the cognitive process of online video selection and provide reference to recommendation research from a psychological perspective.

## 2. Materials and methods

### 2.1. Participants

With informed consent, we recruited 342 participants through online posters: 63.2% female subjects (*n* = 236) and 36.8% male subjects (*n* = 126). The average age was 22 (SD = 2.99), ranging from 18 to 44 (see Table 1 for details). All the participants were users of Bilibili with an account level of two or above, which means that the user has registered and had some experience browsing videos on this platform.

**Table 1:** Demographic information of participants.

**Gender**	**Age**	**Count**
Male	18–26	116
	27–35	9
	36–44	0
	≥45	0
	Missing	1
Female	18–26	205
	27–35	5
	36–44	3
	≥45	0
	Missing	3

### 2.2. Materials

**Figure 1:**
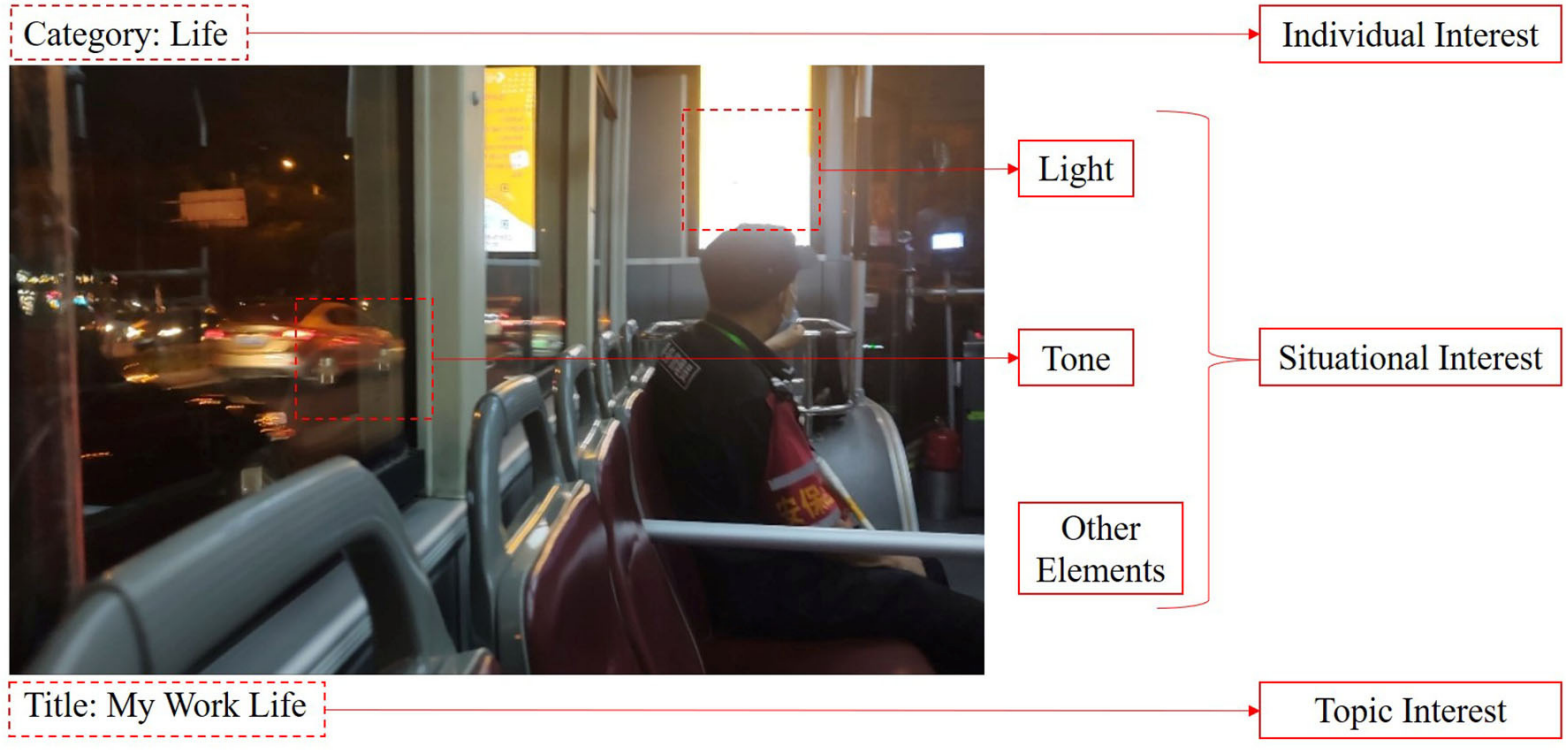
An example of the video elements on the Bilibili website, and the different interests corresponding to them.

Bilibili is an online video-sharing platform similar to YouTube. It is one of the most popular video-sharing platforms in China. The materials of the video category, video title, and video cover presented to the subjects in the experiment were all achieved from this platform. When users browse videos on Bilibili, the video categories are clearly shown on both the top and the sidebar of the page, and the videos are also clustered by their categories on the page. Ten categories (anime, music, dance, game, knowledge, digits, life, otomads, fashion, and entertainment) are featured in Bilibili. Meanwhile, most of the page is occupied by video titles and video covers, which are matched in pairs and shown together. The video cover is a picture containing some visual elements of the video and the video title is located below the picture, stating the specific topic of the video by text (Figure 1). According to the definition of the three interests, the type of video arouses the user's individual interest, and the title of the video arouses the user's topic interest. Since the Bilibili video cover often contains some keywords conspicuously printed on the picture, which also convey some information about the video title, the video cover triggers situational interest and topic interest.

To select the representative videos in the category, the study obtained the required materials from videos with almost 500,000 views within 7 days, which means that the video obtained moderate popularity. In each video category, the earliest six videos that met the criteria were selected as the materials representing the category by randomly refreshing the page, and finally, we obtained 60 videos in total (six for each category). To minimize possible bias while controlling the time cost for a single participant, all the participants were randomly divided into six groups. Each group was randomly assigned one video for each video category during the experiment, which means that the participants in different groups received different experimental materials and the participants in the same group received the same experimental materials.

### 2.3. Measures

#### 2.3.1. Individual interest

A short questionnaire was adapted from a personal interest scale (Ainley, 2006), which was used to measure individual interest to assess the participants' individual interest in a specific video category. It consisted of two items rated on a 5-point Likert scale. Items included the following: “How well do you know this category?” and “How important is this category to you?” A mean score was calculated from the two items, and higher scores meant higher individual interest.

#### 2.3.2. Topic interest

Ainley's Topic Interest Scale measures the level of topic interest by using only one item related to the title (Ainley, 2006). To measure the interest caused by the title in more detail, we added six items to Harakiewicz's Situational Interest Scale (Harakiewicz and Barron, 2008) by changing the questions into ones asking about the video title rather than the situational factors. Finally, our topic interest questionnaire contained seven items. Sample items included the following: “Reading that title, I am interested in the topic presented by that title:” and “Seeing that title, I think the video is interesting:”. A mean score was calculated from the seven items, and higher scores indicated higher topic interest.

#### 2.3.3. Situational interest

Our questionnaire to evaluate situational interest was similar to the above, consisting of the seven items but asking about specific video covers. Sample items included the following: “Seeing the cover of this video, I am interested in the cover:” and “Seeing the cover of this video, I think the video is interesting:” Also, a mean score was calculated from seven items.

#### 2.3.4. Click behavior

The probability of clicking on the video was reported by participants, ranging from 0 (must not click) to 100 (definitely will click).

### 2.4. Procedure

**Figure 2:**
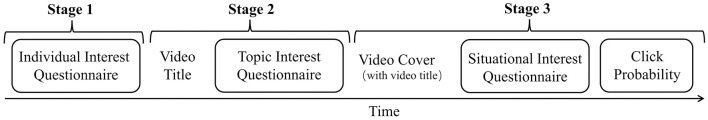
Study procedure for each participant.

The study was conducted through an online webpage containing all the stimuli and questionnaires and made up of three main stages. Each participant would finish three questionnaires (Figure 2). First, 10 video categories and individual interest questionnaires were presented to participants to assess their interests in 10 video categories. Second, 10 video titles and topic interest questionnaires were presented to participants one by one to assess their interest in the 10 video titles. Third, 10 video covers and situational interest questionnaires were presented to participants one by one to assess their interest in 10 video covers.

As participants had already read all the titles in the second stage, and a video cover usually contained varying numbers of keywords of the title, we also presented the corresponding video title closely below each cover picture in the third stage. Considering the participant would process both topic-related and situation-related information in this circumstance, the final situational interest score was calculated by subtracting the topic interest score from the original situation interest score.

### 2.5. Data analysis

To explore the effects of the different interests on click behavior, correlation analysis was first conducted to illustrate their basic relationship. Regression analysis was then used to determine which interest has a greater impact on click behavior. After that, we constructed a mediation model to further analyze the mechanism of interest's influence on click behavior. All these analyses were conducted by Jamovi 2.2.2.

## 3. Results

### 3.1. Correlations

To analyze the relationships between different interests and click probabilities, we first conducted Pearson's correlation analysis on the whole sample, with one individual's response to a video as one case. Then the same analysis was conducted separately in each video category. Taking all samples into consideration, individual interest, topic interest, and situational interest were all significantly correlated with click behavior. In all categories, the relationship between interest and click behavior was consistent with the result of analysis on all samples, and in most categories, the correlation coefficient of the topic interest was higher than the other two interests (see results in Table 2).

**Table 2:** Pearson's correlations among all variables.

**Category**	**Variable**	**Individual interest**	**Topic interest**	**Situational interest**	**Click behavior**
Anime	Individual interest	–	0.28^***^	0.17^**^	0.33^***^
	Topic interest		–	−0.15^***^	0.62^***^
	Situational interest			–	0.48^***^
	Click Behavior				–
Music	Individual interest	–	0.23^***^	−0.04	0.20^***^
	Topic interest		–	−0.35^***^	0.62^***^
	Situational interest			–	0.29^***^
	Click Behavior				–
Dance	Individual interest	–	0.45^***^	−0.08	0.41^***^
	Topic interest		–	−0.34^***^	0.65^***^
	Situational interest			–	0.28^***^
	Click Behavior				–
Game	Individual interest	–	0.48^***^	0.05	0.35^***^
	Topic interest		–	−0.23^***^	0.74^***^
	Situational interest			–	0.25^***^
	Click Behavior				–
Knowledge	Individual interest	–	0.13^*^	−0.002	0.20^***^
	Topic interest		–	−0.36^***^	0.64^***^
	Situational interest			–	0.22^***^
	Click Behavior				–
Digits	Individual interest	–	0.29^***^	0.01	0.25^***^
	Topic interest		–	−0.29^***^	0.65^***^
	Situational interest			–	0.28^**^
	Click Behavior				–
Life	Individual interest	–	0.18^***^	−0.04	0.17^***^
	Topic interest		–	−0.27^***^	0.55^***^
	Situational interest			–	0.47^***^
	Click Behavior				–
Otomads	Individual interest	–	0.34^***^	0.26^***^	0.51^***^
	Topic interest		–	−0.29^***^	0.45^***^
	Situational interest			–	0.56^***^
	Click Behavior				–
Fashion	Individual interest	–	0.50^***^	0.06	0.45^***^
	Topic interest		–	−0.21^***^	0.62^***^
	Situational interest			–	0.42^***^
	Click Behavior				–
Entertainment	Individual interest	–	0.23^***^	0.09	0.25^**^
	Topic interest		–	−0.26^***^	0.71^***^
	Situational interest			–	0.27^***^
	Click Behavior				–
All	Individual interest	–	0.33^***^	0.04^*^	0.32^***^
	Topic interest		–	−0.27^***^	0.66^***^
	Situational interest			–	0.35^***^
	Click Behavior				–

^*^p < 0.05; ^**^p < 0.01; ^***^p < 0.001.

### 3.2. Linear regression

This study utilized the ordinary linear regression method to explore the relationship between different interests and click probability. Different interests were treated as the independent variable, gender of users as the control variable, and click probability as the dependent variable in our analysis. As shown in Table 3, in the whole sample and each category, both topic interest and situational interest significantly positively predicted participants' click behavior. For individual interest, in most categories, the predictive effect on click behavior was no longer significant. The predictive effect of individual interest still existed in the otomads category [*t*_(122)_ = 2.35, *p* = 0.02] for male subjects, in the fashion category [*t*_(212)_ = 2.62, *p* = 0.01] for female subjects, and in the whole sample for female subjects [*t*_(2, 156)_ = 2.81, *p* < 0.01], with a lower regression coefficient than the other two interests. The regression coefficients of topic interest and situational interest also varied in different categories.

**Table 3:** Linear regression for different categories.

**Category**	**Gender**	**Independent variable**	***SE***	**β**	***t***	***P***	***R* ^2^**	***F***	***p***
Anime	Female	Individual interest	0.45	0.02	0.58	0.56	0.76	227.34	<0.001
		Topic interest	0.13	0.75	20.45	<0.001			
		Situational interest	0.13	0.02	18.13	<0.001			
	Male	Individual interest	0.63	−0.06	−1.16	0.25	0.74	115.19	<0.001
		Topic interest	0.17	0.77	15.14	<0.001			
		Situational interest	0.18	0.60	12.46	<0.001			
Music	Female	Individual interest	0.45	0.03	0.99	0.33	0.76	223.99	<0.001
		Topic interest	0.11	0.86	22.88	<0.001			
		Situational interest	0.12	0.72	19.34	<0.001			
	Male	Individual interest	0.69	−0.03	−0.41	0.68	0.76	125.59	<0.001
		Topic interest	0.15	0.92	17.83	<0.001			
		Situational interest	0.19	0.56	11.47	<0.001			
Dance	Female	Individual interest	0.43	0.04	1.14	0.26	0.80	273.78	<0.001
		Topic interest	0.10	0.87	23.54	<0.001			
		Situational interest	0.12	0.57	17.19	<0.001			
	Male	Individual interest	0.83	−0.02	−0.43	0.67	0.75	124.88	<0.001
		Topic interest	0.17	0.91	15.60	<0.001			
		Situational interest	0.17	0.74	14.53	<0.001			
Game	Female	Individual interest	0.45	−0.04	−1.23	0.22	0.80	275.54	<0.001
		Topic interest	0.10	0.93	24.67	<0.001			
		Situational interest	0.13	0.57	17.23	<0.001			
	Male	Individual interest	0.55	−0.05	−1.40	0.16	0.84	216.50	<0.001
		Topic interest	0.12	0.89	22.20	<0.001			
		Situational interest	0.15	0.56	15.16	<0.001			
Knowledge	Female	Individual interest	0.51	0.001	0.06	0.95	0.72	185.72	<0.001
		Topic interest	0.11	0.88	22.05	<0.001			
		Situational interest	0.13	0.64	16.11	<0.001			
	Male	Individual interest	0.59	−0.01	−0.12	0.90	0.67	81.44	<0.001
		Topic interest	0.17	0.92	15.40	<0.001			
		Situational interest	0.18	0.54	9.15	<0.001			
Digits	Female	Individual interest	0.52	−0.01	−0.20	0.84	0.77	230.30	<0.001
		Topic interest	0.12	0.88	22.15	<0.001			
		Situational interest	0.12	0.63	17.68	<0.001			
	Male	Individual interest	0.57	−0.03	−0.76	0.45	0.78	145.30	<0.001
		Topic interest	0.14	0.91	20.12	<0.001			
		Situational interest	0.18	0.49	10.95	<0.001			
Life	Female	Individual interest	0.51	0.06	1.86	0.06	0.76	226.46	<. 001
		Topic interest	0.11	0.79	21.14	<0.001			
		Situational interest	0.12	0.74	20.28	<0.001			
	Male	Individual interest	0.63	−0.03	−0.61	0.54	0.79	154.46	<0.001
		Topic interest	0.14	0.83	18.78	<0.001			
		Situational interest	0.15	0.69	15.81	<0.001			
Otomads	Female	Individual interest	0.52	0.06	1.69	0.09	0.78	243.97	<0.001
		Topic interest	0.13	0.69	17.08	<0.001			
		Situational interest	0.11	0.81	21.24	<0.001			
	Male	Individual interest	0.67	0.11	2.35	0.02	0.79	149.52	<0.001
		Topic interest	0.16	0.74	14.63	<0.001			
		Situational interest	0.16	0.76	15.45	<0.001			
Fashion	Female	Individual interest	0.48	0.10	2.62	0.01	0.79	257.46	<0.001
		Topic interest	0.11	0.75	19.14	<0.001			
		Situational interest	0.12	0.64	18.75	<0.001			
	Male	Individual interest	0.71	0.002	0.09	0.93	0.78	141.99	<0.001
		Topic interest	0.15	0.83	16.19	<0.001			
		Situational interest	0.19	0.61	13.59	<0.001			
Entertainment	Female	Individual interest	0.49	0.01	0.37	0.71	0.77	238.94	<0.001
		Topic interest	0.11	0.90	23.20	<0.001			
		Situational interest	0.13	0.60	19.40	<0.001			
	Male	Individual interest	0.57	0.02	0.54	0.59	0.80	159.64	<0.001
		Topic interest	0.13	0.88	20.61	<0.001			
		Situational interest	0.19	0.52	12.28	<0.001			
All	Female	Individual interest	0.14	0.03	2.81	0.01	0.78	2504.03	<0.001
		Topic interest	0.03	0.83	70.73	<0.001			
		Situational interest	0.04	0.65	59.03	<0.001			
	Male	Individual interest	0.19	−0.01	−0.46	0.64	0.77	1389.39	<0.001
		Topic interest	0.04	0.87	57.00	<0.001			
		Situational interest	0.05	0.62	42.53	<0.001			

### 3.3. Mediation analysis

The results of correlation analysis showed the facilitative effects of all three interests on click behavior, while individual interest was not significant in the linear regression model in many video categories. To further explore how different types of interests affect click behavior in different video categories, we conducted mediation analyses. As one video category contains mass-specific videos, topic interest and situational interest triggered by a specific video may have more direct effects than the individual interest triggered by video categories. In this step, we explored whether topic interest and situational interest mediate the effect of individual interest on click behavior.

For all samples, the topic interest partly mediates the effect of individual interest on click behavior which means the direct effect and the indirect effect is significant. Situational interest's mediating effect is not significant in statistics. The mediation analysis results also vary across categories. According to the relationship between individual interest and click behavior, the analysis results can be divided into two main types: partly mediating effect and full mediating effect (see results of each category in Table 4).

**Table 4:** Mediation analysis for all categories.

**Categories**	**Variables**	**Click behavior**		**Topic interest**		**Situational interest**	
		β	***t***	β	***t***	β	***t***
Anime	Individual interest	−0.004	−0.153	0.28	5.39	0.19	3.50
	Topic interest	0.67	26.98				
	Situational interest	0.56	23.19				
Music	Individual interest	0.01	0.64	0.24	4.57	−0.01	−0.26
	Topic interest	0.73	32.06				
	Situational interest	0.55	24.77				
Dance	Individual interest	0.02	0.64	0.46	9.54	−0.03	−0.56
	Topic interest	0.74	30.79				
	Situational interest	0.54	24.98				
Game	Individual interest	−0.03	−1.26	0.47	9.91	−0.09	−1.71
	Topic interest	0.82	35.10				
	Situational interest	0.50	24.17				
Knowledge	Individual interest	0.003	0.10	0.11	2.203	−0.02	−0.415
	Topic interest	0.74	30.06				
	Situational interest	0.49	20.15				
Digits	Individual interest	−0.01	−0.46	0.27	5.28	0.03	0.52
	Topic interest	0.76	33.01				
	Situational interest	0.50	22.52				
Life	Individual interest	0.02	0.73	0.20	3.78	0.02	0.29
	Topic interest	0.68	30.40				
	Situational interest	0.61	27.85				
Otomads	Individual interest	0.07	3.19	0.34	6.66	0.24	4.49
	Topic interest	0.57	26.08				
	Situational interest	0.65	30.56				
Fashion	Individual interest	0.05	2.02	0.50	10.81	0.03	0.56
	Topic interest	0.69	26.84				
	Situational interest	0.55	25.00				
Entertainment	Individual interest	0.01	0.52	0.24	4.60	0.08	1.519
	Topic interest	0.77	35.20				
	Situational interest	0.49	22.89				
All	Individual interest	0.05	2.42	0.33	20.62	0.01	1.85
	Topic interest	0.72	97.97				
	Situational interest	0.54	78.43				

**Figure 3:**
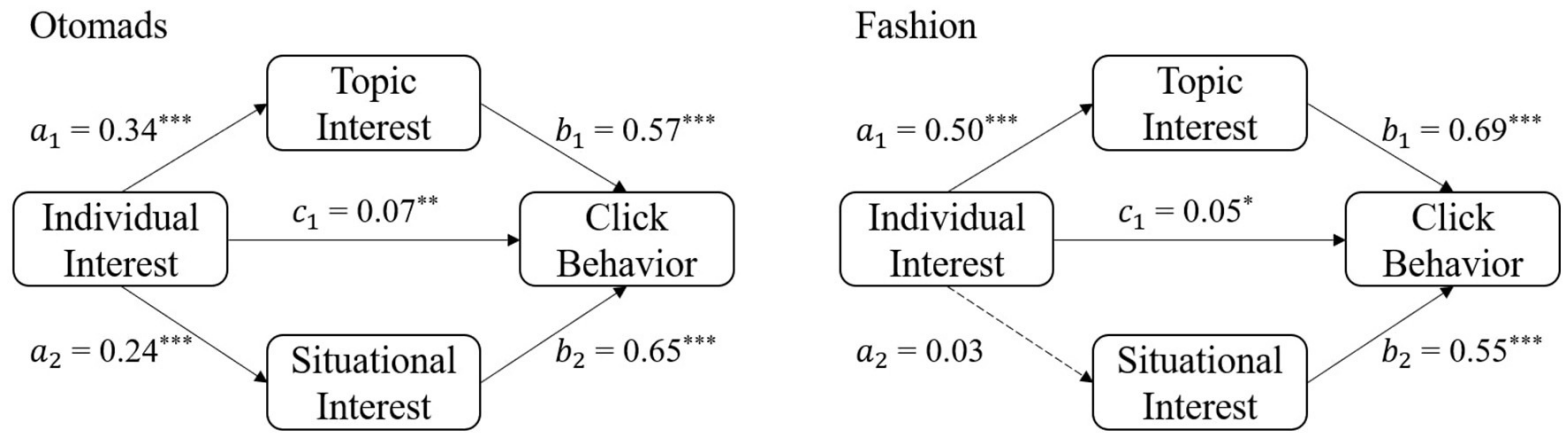
Mediation effect of topic interest and situational interest on individual interest and click behavior in the otomads and fashion categories.

In the otomads and fashion categories, the relationship between individual interest and click behavior is partly mediated (Figure 3). More specifically, topic interest and situational interest have a partial mediation effect between individual interest and click behavior in the otomads category. In the fashion category, only the topic interest has a partial mediation effect between individual interest and click behavior.

**Figure 4:**
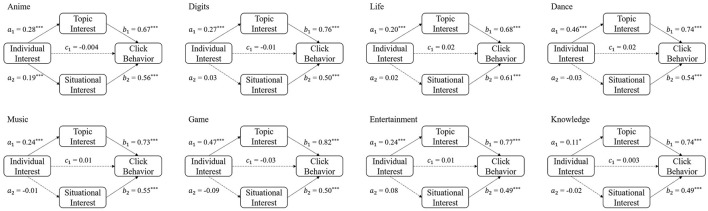
Mediation effect of topic interest and situational interest on individual interest and click behavior in the anime, digits, life, dance, music, game, entertainment, and knowledge categories.

In the anime, digits life, dance, music, game, entertainment, and knowledge categories, the relationship between individual interest and click behavior is fully mediated (Figure 4). More specifically, topic interest and situational interest have a full mediation effect between individual interest and click behavior in the anime and game categories. In the digits, life, dance, music, entertainment, and knowledge categories, only topic interest plays a full mediation effect.

## 4. Discussion

Through correlation analysis, regression analysis, and mediation analysis, we explored the role of individual interest, topic interest, and situational interest on click behavior in online video browsing situations. For all samples and each category, the results of correlation analysis showed that individual interest, topic interest, and situational interest all had a significant positive relationship with click behavior. All three interests had a facilitative effect on click behavior, which supported our first preliminary hypothesis. This result was also consistent with the previous studies showing the effects of interest on learning (Ainley et al., 2002). Generally, topic interest showed a markedly higher correlation with click behavior, while the correlation coefficients between individual interest and click behavior were close to the correlation coefficients between situational interest and click behavior. This result indicated that individual interest and situational interest may have a similar predictive power for click behavior, and the predictive power of topic interest was higher than theirs. However, in the regression analysis results, the contribution of individual interest in predicting click behavior was not significant in most of the video categories, while the contribution of situational interest still existed. This result implied that the predictive relationship between individual interest and click behavior may be mediated by topic interest or situational interest in many video categories. As the predictive effects of the three interests showed differences among different video categories, mediation analysis was conducted separately on each video category to further clarify the mechanism of individual interest in predicting click behavior.

The mediation analysis further revealed the influence pattern of the three interests on click behavior, and there were mainly two different patterns for different categories of videos.

### 4.1. Partially mediated individual-interest effect

For the otomads and fashion categories, users' interest in the category did have an impact on the possibility of them clicking on the video. Users who follow these categories were more likely to click on videos in these categories, and at least part of that effect was independent of the title and cover of the video. This means that even though the specific content of the video does not really interest the user, there is a certain possibility that the user will click on the video. In these categories, the user's internal needs and knowledge reflected by individual interest played a role in facilitating the further exploration of a video, which was consistent with the role the individual interest played in learning studies (Fransson, 1977; Zhang and Zhang, 1996). Meanwhile, the effect of individual interest was also mediated by topic interest and situational interest to a large extent, showing the greater effect of the other two interests in triggering clicks.

### 4.2. Fully mediated individual-interest effect

For the anime, digits, life, dance, music, game, entertainment, and knowledge categories, the effect of individual interest was fully mediated by topic interest or situational interest, or both. The user's individual interest in these domains did affect the probability of clicking on a video in these categories, but that was essential because the domain interest predicted the interest in a specific video title or a specific video cover in this category. The topic interest and situational interest finally triggered click behavior, which means that for these categories, users might not click on a video if the specific content of the video does not interest them.

Considering the results of all analyses together, we found that three types of interest had different predictive power on online video click behavior and revealed how they have an impact on click behavior, which supported the second hypothesis. In most video categories, topic interest plays the most important role in predicting click behavior, which means that the cognitive processing of the video title will greatly affect the occurrence of subsequent exploration behavior (Prestwich et al., 2010). This result implied that if we want to attract users to click on a video, the most effective way would be matching the video title to the users' topic interest. For video recommendation, it could be most important to find the topics that users are interested in. Situational interest is also of great significance. A previous study showed that the richer the visual stimuli, the more willing the individual was to engage in subsequent exploration behavior (Hsieh et al., 2012). In our study, a video cover that can evoke users' interest in visual elements can also attract users to watch the video. For video recommendation, in addition to the topic interest caused by the video title, discovering and recommending videos that match users' interests in the visual elements of the cover may also have a significant effect. In particular, when there are multiple videos around the same topic, the situational interest evoked by the visual element is likely to play an important role in determining which video a user would click on.

Individual interest also has a significant effect on click behavior, which implies that the user's inherent preference and tendency will predict further exploration behavior (Perera and Zimmermann, 2017). Meanwhile, the predictive effect of the individual is largely mediated by topic interest or situational interest. However, individual interests are still important for predicting click behavior. First, when users browse video websites, they often directly enter the page of a certain category, and the user's individual interest undoubtedly plays a very important role in such a category selection process. Second, our result showed that individual interest has a strong relationship with topic interest and situational interest. If topic interest and situational interest are hard to obtain, the individual interest would be a useful indicator of the other two interests as well as click behavior.

The differences in the result patterns in different categories also provide us with more knowledge about the specific effects of interest. The results in the otomads category showed that situational interest was more important in predicting click behavior than topic interest. The reason for this phenomenon needs to be investigated from the characteristics of the video in the otomads category. Otomads refers to a kind of adapted video that subverts the classics, deconstructs the tradition, promotes the personality, strengthens the focus, and satirizes the society by dissecting social hot issues and then repeating and re-creating them. The video cover in otomads is often an adaption of the original video material to make it more entertaining. Thus, a prominent feature of the video covers in otomads is the use of richer colors and more exaggerated stickers, which could attract more attention from users. This may explain why situational interest in the otomads category plays a greater role in predicting click behavior.

The mediation analysis in the otomads and fashion categories showed that individual interest not only promoted click behavior through situational interest or topic interest but also directly promoted click behavior. Such a direct effect was not found in other categories. This may be because the videos in the otomads and fashion categories are distinctive features that attract fans no matter what the specific video content is. Meanwhile, these features should be quite easy to recognize. Thus, the fans of the two categories could identify the otomads or fashion videos from the title and cover, and their desire to exploration could be aroused just by this fact.

The effect of individual interest was mediated by topic interest in all categories but mediated by situational interest only in the otomads and anime categories. This means that individual interest can only have an impact on click behavior through situational interest in these two categories. The reason for this result may be that understanding the video cover content in these two categories requires some prior knowledge, which is not easy to obtain in daily life. The users with high individual interest have more prior knowledge about the content of the categories, so they could understand the video covers more easily. For those with low individual interest and less or no prior knowledge, they cannot fully process the visual elements in those covers.

It should also be noted that the difference in the predictive powers of individual interest among categories may be caused by different reasons. One possible reason is that the impact of individual interest on click behavior really is different among categories. Another possible influencing factor is the difference in how a video title and cover reflect the category of the video. Although for most categories the title and cover could obviously show the video category, sometimes the title and cover may reflect the category information less clearly in some categories. When users processed titles and covers of different categories in our experiment, the difference in their capabilities of reflecting their categories may also lead to variations in the predictive power of individual interest. Thus, it is reasonable to be more cautious in understanding the difference in the predictive powers of individual interest among different categories.

Today, the information overload prevalent on the internet means it is extremely valuable to get users' attention (Renjith, 2017), and clicking to watch is a typical behavior, indicating that users do pay attention to and process something. As the purpose of recommending is usually to induce users to click for further exploration, the roles of different interests and their influence patterns on click behavior could shed some light on the improvement of the recommender. Recommenders are trying to make use of a variety of information, such as past subscriptions, search history, and demographic information. It is always a crucial question of what information is more important to predict users' clicks (Isinkaye et al., 2015). The historical click record was typically used directly as the label to train data-driven predictive models to recommend videos similar to those the user clicked on previously. Without knowing how interest affects click behavior, these recommendation models may lead to excessive homogeneity of the recommended results and a lack of comprehensibility of the recommending process (Zhang and Chen, 2020). Our study revealed the different effects of the three interests on click behavior among categories, and different interests were triggered by different information channels (i.e., the category, the title, and the cover image) of the video platform. These findings could help to make use of certain information pertinently to improve the effectiveness of recommendation in different categories. The recommender could also try to focus on one certain interest in order to recommend the videos that are able to obtain clicks from users but not that similar to their previous records, expanding the richness of recommendations. If the users' interests could be obtained by tags, records, or even questionnaires, the predictive power of different interests in our results could also directly guide the recommendation, in combination with the category and content information of videos. Meanwhile, the knowledge of the relationships between users' different interests and final choices could not only help to improve the performance of the recommender but also make it more understandable. In addition to the recommendation, attracting users' attention is actually the goal of many stakeholders, and their difficulties in obtaining and using the information from different channels of a platform vary a lot. Understanding the relationships between different interests and clicks may also help service operators, content producers, and advertisers to better choose the information channels and achieve their goals.

As a preliminary study, there are also some limitations that need to be noted. Although we have revealed the influence mechanism of different interests on click behavior, the reasons for the inconsistency of influence patterns in various categories are still unclear. This requires further analysis of the differences among video categories. Different categories of videos may differ in the “focus area” when they were produced (Bopp et al., 2019), e.g., the video producers in the entertainment category may focus more on the exquisite design of the title and cover, while those in the knowledge category may not. On the other hand, the state of users, such as the cognitive workload, may also be different while browsing videos of different categories. It should also be noted that the users of Bilibili are more youthful and more likely to be female, which may limit the generalizability of the findings to other populations to some extent. In addition, our study was also restricted by the features and settings of Bilibili, and it is important to be careful when generalizing our findings to other social media. Focusing on the video category, title, and cover, we did not present and explore other elements possibly influencing click preference, such as the length of the video, which could be investigated in future studies. Restricted by the capacity of one experiment, we could use limited video materials for each category, and in future studies, researchers could expand the video sample to certain categories for better generalizability. In addition, the difference in the degree to which the covers and titles reflect the category could possibly affect the correlation of individual interests, which is also worth verifying in future studies.

In conclusion, we found that the theoretical division of interest in educational psychology could also be applicable to online video browsing situations. Individual interest, topic interest, and situational interest can promote click behavior to different degrees when users browse online videos, and topic interest generally has the greatest impact except for the otomads category. Topic and situational interests partially or completely mediated the effect of individual interests on click behavior, and the mediation effect varied among different categories. Our results could provide psychological perspectives of interest and click behavior to the recommendation and other relevant applications in the online social media scenario.

## Data availability statement

The raw data supporting the conclusions of this article will be made available by the authors, without undue reservation.

## Ethics statement

The studies involving human participants were reviewed and approved by the Institute of Psychology, Chinese Academy of Sciences. The patients/participants provided their written informed consent to participate in this study.

## Author contributions

NZ contributed to the conception, the design of the study, and the manuscript writing. DL collected the data, performed most of the statistical analysis, and wrote the manuscript with input from all authors. ZT conducted part of the experiment and performed part of the statistical analysis. All authors contributed to the article and approved the submitted version.
