# Supplementary figures and images for: How does users' interest influence their click behavior?: evidence from Chinese online video media
Source: Front Psychol. 2023 Jul 6;14:1101396. doi: 10.3389/fpsyg.2023.1101396 (PMC10359899; doi:10.3389/fpsyg.2023.1101396)

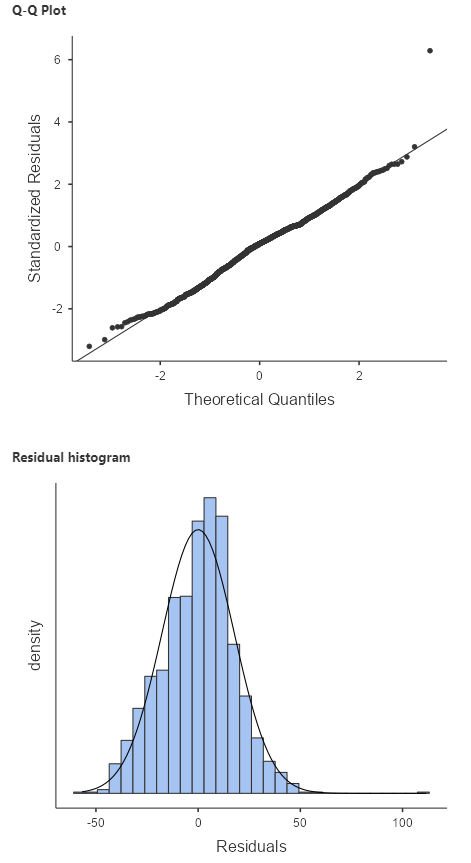

Supplement: Supplementary file 1 [file Data_Sheet_1.ZIP › Test for liner regression/all_female.png]

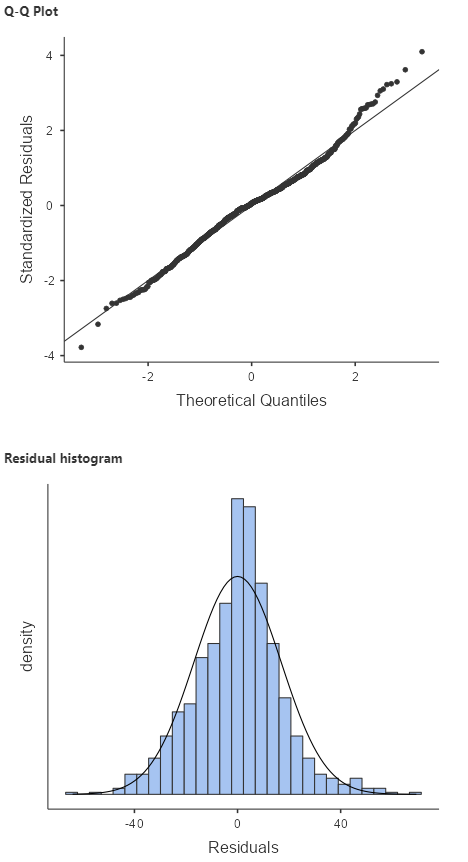

Supplement: Supplementary file 1 [file Data_Sheet_1.ZIP › Test for liner regression/all_male.png]

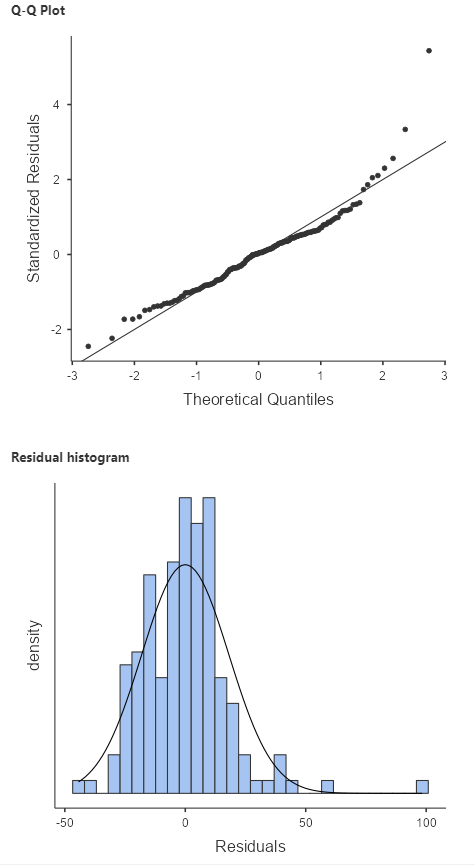

Supplement: Supplementary file 1 [file Data_Sheet_1.ZIP › Test for liner regression/anime_female.png]

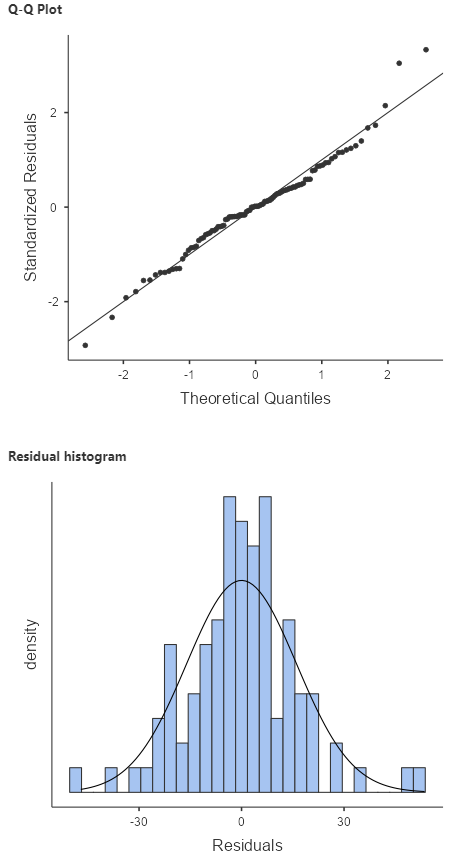

Supplement: Supplementary file 1 [file Data_Sheet_1.ZIP › Test for liner regression/anime_male.png]

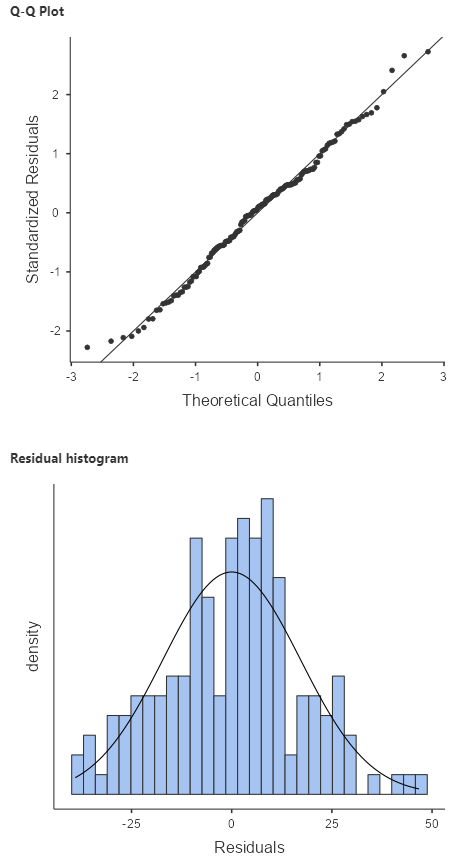

Supplement: Supplementary file 1 [file Data_Sheet_1.ZIP › Test for liner regression/dance_female.png]

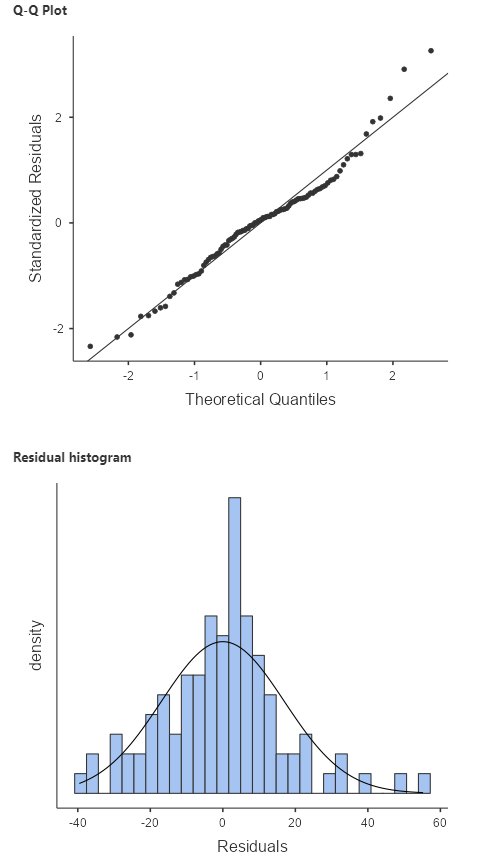

Supplement: Supplementary file 1 [file Data_Sheet_1.ZIP › Test for liner regression/dance_male.png]

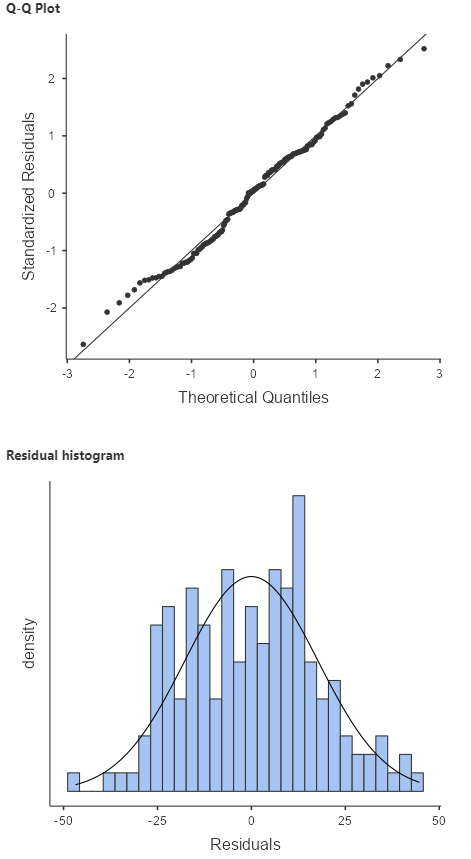

Supplement: Supplementary file 1 [file Data_Sheet_1.ZIP › Test for liner regression/digits_female.png]

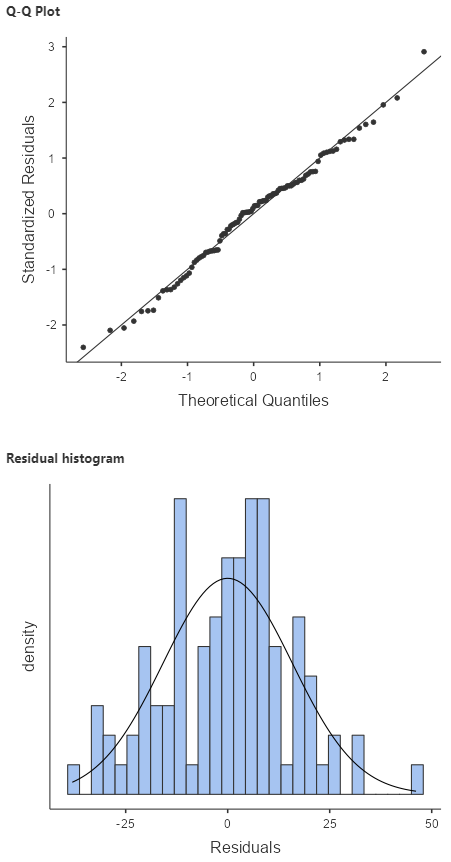

Supplement: Supplementary file 1 [file Data_Sheet_1.ZIP › Test for liner regression/digits_male.png]

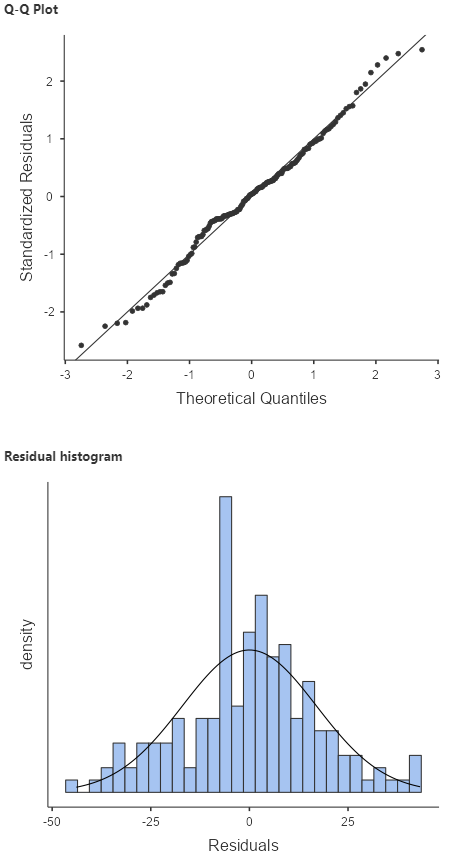

Supplement: Supplementary file 1 [file Data_Sheet_1.ZIP › Test for liner regression/entertainment_female.png]

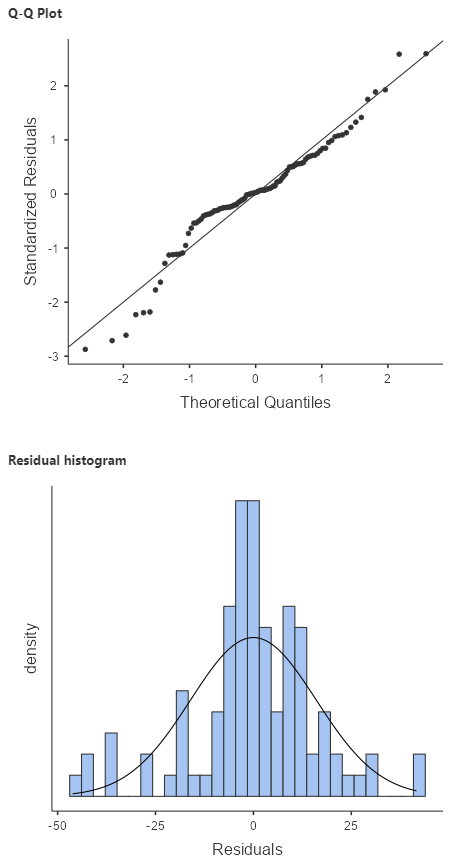

Supplement: Supplementary file 1 [file Data_Sheet_1.ZIP › Test for liner regression/entertainment_male.png]

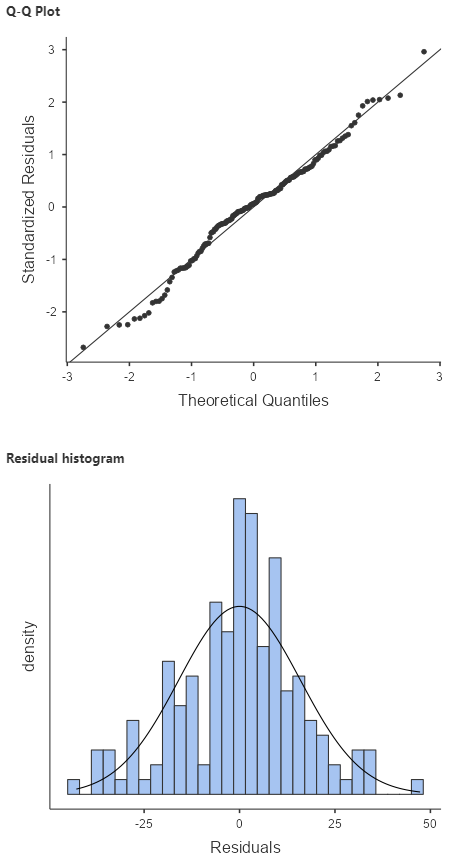

Supplement: Supplementary file 1 [file Data_Sheet_1.ZIP › Test for liner regression/fashion_female.png]

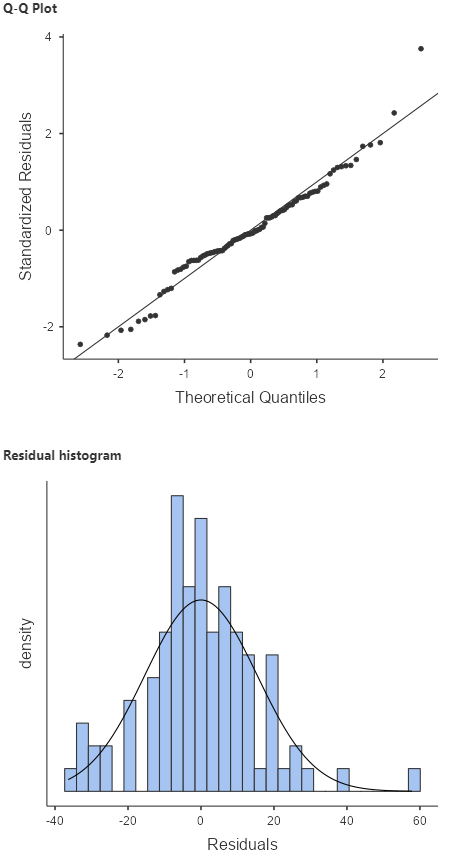

Supplement: Supplementary file 1 [file Data_Sheet_1.ZIP › Test for liner regression/fashion_male.png]

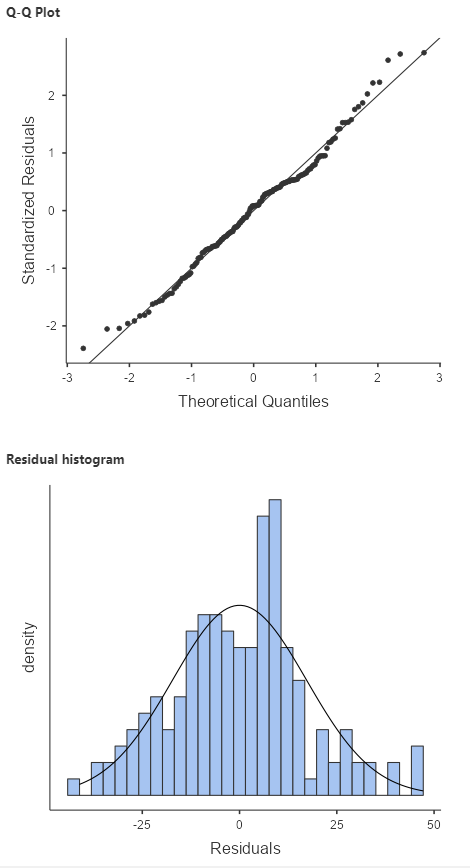

Supplement: Supplementary file 1 [file Data_Sheet_1.ZIP › Test for liner regression/game_female.png]

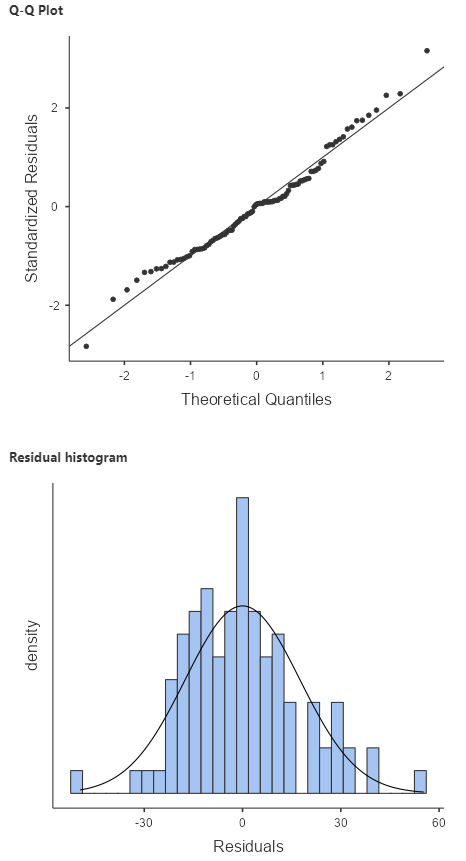

Supplement: Supplementary file 1 [file Data_Sheet_1.ZIP › Test for liner regression/game_male.png]

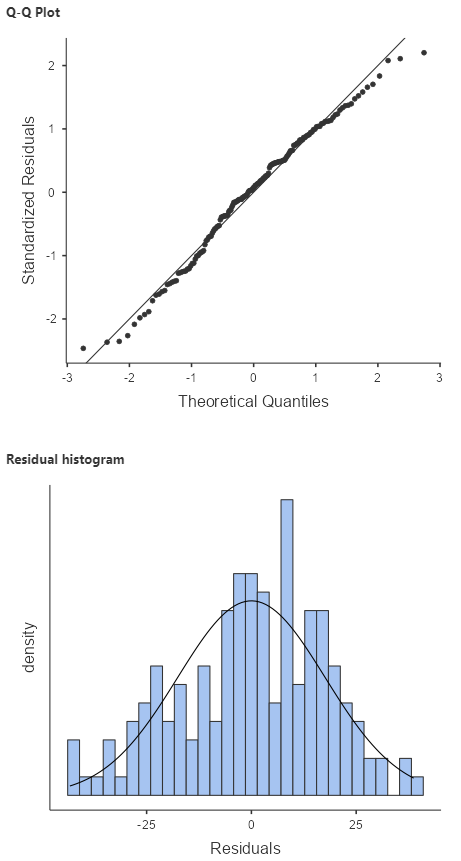

Supplement: Supplementary file 1 [file Data_Sheet_1.ZIP › Test for liner regression/knowledge_female.png]

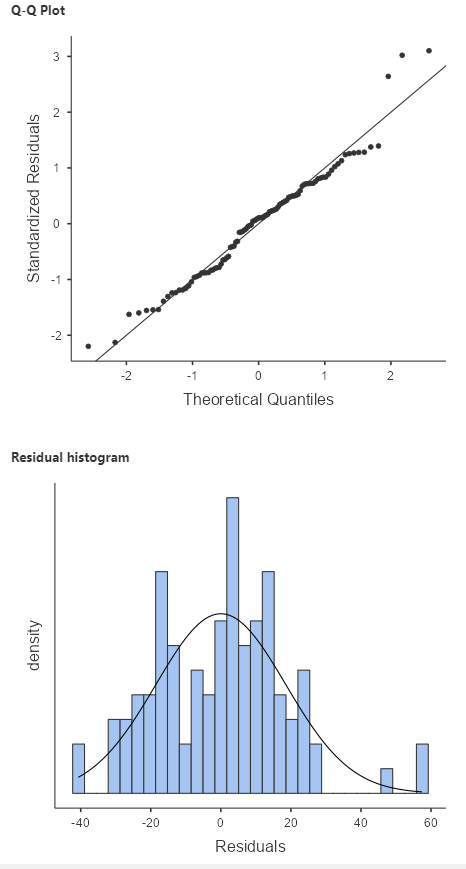

Supplement: Supplementary file 1 [file Data_Sheet_1.ZIP › Test for liner regression/knowledge_male.png]

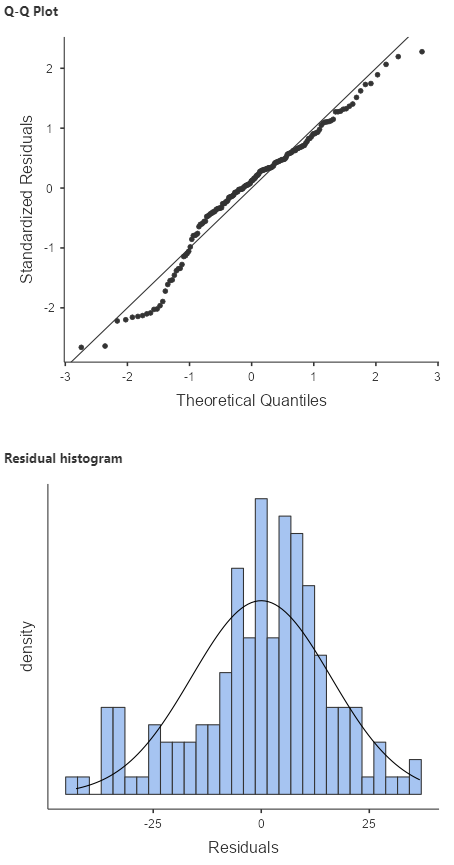

Supplement: Supplementary file 1 [file Data_Sheet_1.ZIP › Test for liner regression/life_female.png]

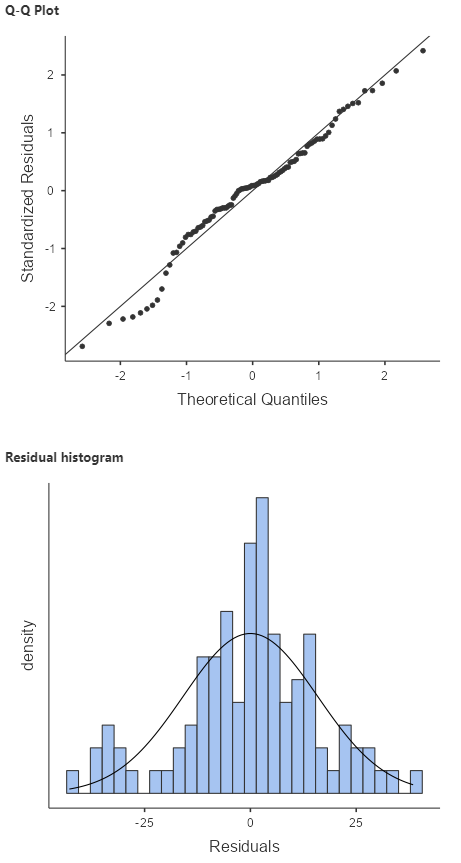

Supplement: Supplementary file 1 [file Data_Sheet_1.ZIP › Test for liner regression/life_male.png]

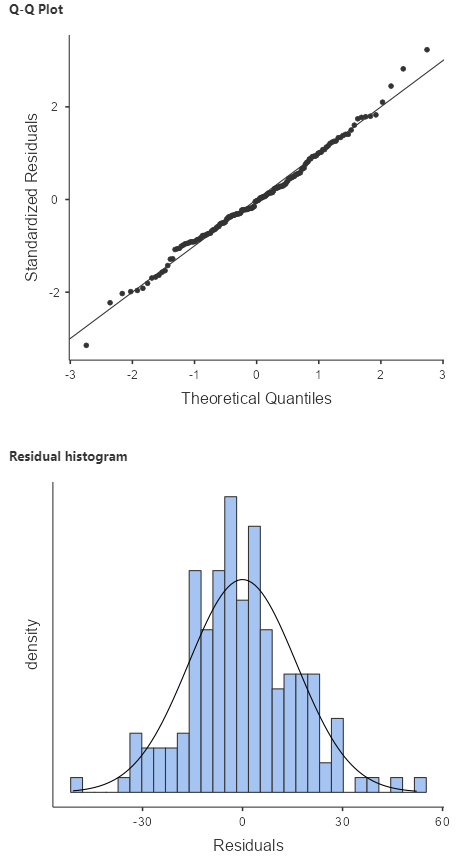

Supplement: Supplementary file 1 [file Data_Sheet_1.ZIP › Test for liner regression/music_female.png]

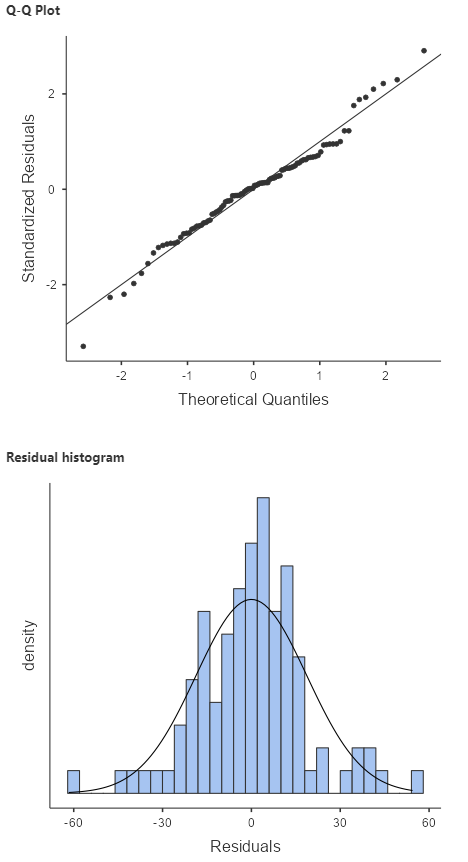

Supplement: Supplementary file 1 [file Data_Sheet_1.ZIP › Test for liner regression/music_male.png]

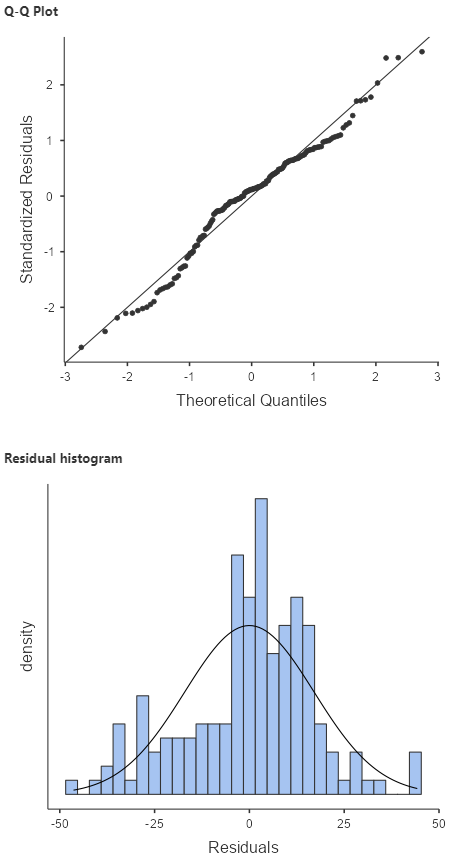

Supplement: Supplementary file 1 [file Data_Sheet_1.ZIP › Test for liner regression/otomads_female.png]

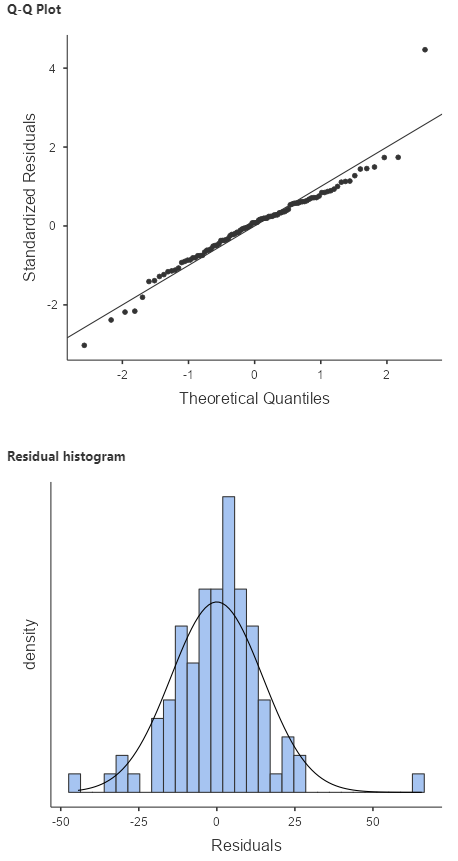

Supplement: Supplementary file 1 [file Data_Sheet_1.ZIP › Test for liner regression/otomads_male.png]
